# *Olig1/2* Drive Astrocytic Glioblastoma Proliferation Through Transcriptional Co-Regulation of Various Cyclins

**DOI:** 10.3390/genes16050573

**Published:** 2025-05-13

**Authors:** Yu Tian, Ziwu Wang, Mengge Sun, Jialin Li, Wenhui Zheng, Feihong Yang, Zhuangzhi Zhang

**Affiliations:** 1 State Key Laboratory of Medical Neurobiology and MOE Frontiers Center for Brain Science, Institutes of Brain Science, Department of Neurology, Fudan University, Shanghai 200032, China; 22111520041@m.fudan.edu.cn (Y.T.); 21111520035@m.fudan.edu.cn (Z.W.); 20111520026@fudan.edu.cn (M.S.); 22111210061@fudan.edu.cn (J.L.); 23211520061@m.fudan.edu.cn (W.Z.); 2 Department of Anesthesiology, Shanghai University of Traditional Chinese Medicine, Shanghai 201203, China; 22022282@shutcm.edu.cn

**Keywords:** glioblastoma (GBM), *Olig1*, *Olig2*, transcriptional regulation

## Abstract

As the most aggressive primary brain tumor, glioblastoma (GBM) is considered incurable due to its molecular heterogeneity and therapy resistance. Identifying key regulatory factors in GBM is critical for developing effective therapeutic strategies. Based on the analysis of TCGA data, we confirmed a robust co-expression and correlation of OLIG1 and OLIG2 in human GBM. However, their roles in the astrocytic GBM subtype remain unclear. In this study, we first establish an astrocytic-featured GBM mouse model by introducing PiggyBac-driven hEGFRvIII plasmids and demonstrate that both OLIG1 and OLIG2 are highly expressed within this context. Next, using CRISPR/Cas9 technology to knockout *Olig1/2*, we found that astrocyte differentiation markers such as GFAP, SOX9, and HOPX were preserved, but tumor cell proliferation was significantly diminished. Mechanistically, CUT&Tag-seq revealed that OLIG1/2 directly binds to the promoter region of various cyclins (*Cdk4*, *Ccne2*, *Ccnd3*, and *Ccnd1*), where an enrichment of the active histone marker H3K4me3 was observed, indicating transcriptional activation of the genes. Notably, *Olig1/2* knockout did not suppress tumor initiation or migration, suggesting that their primary role is to amplify proliferation rather than to drive tumorigenesis. This study defines *Olig1* and *Olig2* as master regulators of GBM proliferation through various cyclins, thereby offering a novel therapeutic target.

## 1. Introduction

Glioblastoma (GBM), recognized as the most aggressive primary brain tumor, is characterized by its profound molecular heterogeneity and therapy resistance. Although advances in genomic profiling have revealed distinct glioblastoma subtypes—proneural, classical, neural, and mesenchymal [1,2]—the underlying mechanisms driving tumor progression across these subtypes remain incompletely understood.

Among the key transcription factors, *Olig1* and *Olig2* are critical for oligodendrocyte lineage development [3,4,5,6,7,8,9,10,11]. Intriguingly, both factors are highly expressed in multiple cancers, including GBM [1,12,13,14,15,16]. Previous studies have predominantly focused on *Olig2*, highlighting its roles in the reprogramming of GBM cells, resistance to genotoxic therapy, and the plasticity of tumor phenotypes [17,18,19,20]. However, the co-expression patterns of *Olig1* and *Olig2* suggest potential synergistic contributions to malignant progression.

Our recent research has demonstrated that *Olig1* and *Olig2* function cooperatively to regulate glial cell development and are critically involved in the initiation of proneural glioma [21,22]. Notably, while the function of *Olig1* and *Olig2* has been extensively studied in a proneural GBM subtype characterized by oligodendroglial features [23,24,25,26], their functional significance in classical GBM (astrocytic signature)—a clinically prevalent and aggressive variant [27]—remains unexplored. This knowledge gap underscores the need to dissect their cooperative mechanisms in astrocytic-featured GBM contexts.

In this study, we hypothesize that *Olig1/2* collaboratively drive astrocytic GBM progression by regulating the proliferation of tumor cells. To test this, we integrated multi-omics analyses, conditional knockout models, and dual-reporter systems to unravel their roles in astrocytic-featured GBM. We validated their co-expression and correlation in human GBM cohorts, then established a mouse model recapitulating astrocytic GBM pathology. By coupling CRISPR/Cas9-mediated double knockout with lineage tracing, we found that the loss of *Olig1/2* does not alter glioma characteristics but inhibits tumor proliferation. Furthermore, we mechanistically linked their oncogenic activity to direct transcriptional activation of various cyclins, the key drivers of cell proliferation, by CUT&Tag-seq analysis.

Our findings not only redefine *Olig1/2* as synergistic regulators of astrocytic GBM proliferation but also unveil their conserved roles in cell cycle regulation that is critical for tumor growth. This work provides a framework for targeting *Olig1/2*-dependent pathways in GBM and highlights the importance of context-specific transcriptional networks in gliomagenesis.

## 2. Materials and Methods

### 2.1. Animals

All experiments conducted in this study were in accordance with guidelines from Fudan University (No. 20220228-140). The generation and genotyping of the *IS*-reporter [28] and *H2b*-reporter mice [28] were as described previously. The *Olig1/2^F/+^* mouse was generated using the CRISPR/Cas9 strategy, in which Lox2272 sites were inserted into non-conserved regions upstream of the 5′UTR and downstream of the 3′UTR of the Olig2 gene, enabling the conditional knockout of exons 1 and 2. Through a secondary targeting strategy, Loxp sites were inserted 2 kb upstream of the 5′UTR and downstream of the 3′UTR of the Olig1 gene, specifically targeting its single exon [22]. Wild-type mice or littermates without the *Cre* allele were used as controls. All mice were maintained on a mixed genetic background of C57BL/6J and CD1. The mice were allowed access to water and food ad libitum and maintained on a 12-h light/dark cycle. The day of vaginal plug detection was considered embryonic day 0.5 (E0.5), and the day of birth was defined as postnatal day 0 (P0). Both male and female mice were used in all experiments.

### 2.2. GBM Mouse Model Generation

At P0, plasmids encoding PBase, hEGFRvIII, and Cre were delivered into the *H2b*-reporter or *IS*-reporter mice via IUE. The amount of plasmid used for each electroporation was constant.

### 2.3. Plasmid Construction

The *pCAG-Cre* vector was constructed by our laboratory. In the *pCAG-Cre* vector, the coding sequence of Cre was replaced with PiggyBac transposase to generate the pCAG-PiggyBac transposase vector. For tumor-inducing constructs, hEGFRvIII was cloned downstream of the CAG promoter, and GFP was replaced with HA-tagged dominant-negative p53 (dnP53) [29].

### 2.4. In Utero Electroporation

The plasmid solution containing DNA (1.5–2 mg/mL) and 0.05% Fast Green dye was precisely injected into the cerebral ventricles of P0 mice using a glass needle (0.5 μL per ventricle), ensuring that the amount of plasmid injected each time was consistent. Subsequently, electroporation was performed using a square wave electroporator (ECM830, BTX, Holliston, MA, USA) with parameters set to 100 volts, 5 pulses (50 ms per pulse, 950 ms intervals).

### 2.5. Immunofluorescence Staining

Immunofluorescence staining was performed as previously described [30]. Briefly, brain sections were thoroughly washed with 0.05 M TBS, followed by high-temperature antigen retrieval (boiling for 20 min) to expose epitopes. Subsequently, sections were permeabilized with 0.5% Triton ×-100 for 30 min to enhance antibody penetration and blocked with 5% donkey serum for 2 h to reduce nonspecific binding. Primary antibodies were incubated overnight at 4 °C. After three TBS washes, corresponding secondary antibodies were applied and incubated at room temperature for 2 h in the dark. Nuclei were counterstained with DAPI (1 μg/mL) for 1–3 min. Sections were mounted with antifade mounting medium, stored at 4 °C in the dark, and imaged using a fluorescence microscope.

We used the following primary antibodies: GFP (1:5000, chicken, Aves Labs GFP-1020), tdTomato (1:2000, goat, SICGEN Ab8181), MKI67 (1:1000, mouse, BDPharmingen 556003), OLIG2 (1:1000, rabbit, Oasis OB-PRB009), OLIG1 (1:1000, rabbit, Oasis N/A), SOX10 (1:500, goat, Oasis OB-PGP001), GFAP (1:1500, rabbit, DAKO Z0334), SOX9 (1:2000, rabbit, Abcam ab185230), HOPX (1:500, rabbit, Oasis OB-PRT015), CASPASE3 (1:1000, rabbit, Abcam ab32351).

### 2.6. GEPIA

Gene Expression Profiling Interactive Analysis (GEPIA), according to databases of TCGA and GTEx, offers crucial customizable and interactive functions like dimensionality reduction analysis, similar gene detection, correlation analysis, patient survival analysis, profiling plotting, and differential expression analysis [31]. Utilizing GEPIA, this study explored the expression of OLIG1 and OLIG2 in different types of GBM tumor tissues and controls through box plots. Genes with |log2FC| values higher than 1 and *p*-values below 0.05 were classified as differentially expressed genes.

### 2.7. UALCAN

UALCAN, based on CBTTC, CPTAC, MET500, and TCGA, is a comprehensive online tool for in-depth studies of cancer OMICS data [32]. Utilizing UALCAN, this study explored the expression of OLIG1 and OLIG2 across TCGA cancers and controls through box plots and explored the correlation analysis of OLIG2 and OLIG1 expression in GBM.

### 2.8. TIMER

The TIMER database is a comprehensive resource that can be used to evaluate the immune efficacy of various types of cancer systematically [33]. We used TIMER to analyze the correlation between OLIG1 and OLIG2 infiltrating immune cells (CD4+  T cells, B cells, CD8+  T cells, neutrophils, dendritic cells, and macrophages) in GBM. The correlation between immune characteristic genes and immune checkpoints was studied through Spearman correlation analysis. A value with *p*  <  0.05 is considered statistically significant, and the correlation coefficient’s absolute value is close to 1, indicating a stronger correlation. SCNA modules are used for the comparison of tumor infiltration levels among tumors with different somatic copy number alterations for OLIG1 and OLIG2. Box plots are presented to show the distributions of each immune subset at each copy number status in selected cancer types. The infiltration level for each SCNA category is compared with the normal using a two-sided Wilcoxon rank sum test.

### 2.9. CUT&Tag-Seq

CUT&Tag-seq was performed as previously described [34] using the Vazyme TD901 kit(VAZYME, Nanjing, China). Cells were bound to Concanavalin A-coated beads, incubated with primary antibodies (1 μg Rabbit-anti-OLIG2/Rat-anti-OLIG1 for the experimental group; no antibody for control), followed by species-matched secondary antibodies. After Tn5 transposase (TTE mix) treatment and DNA tagmentation, chromatin fragments were purified through phenol-chloroform extraction and ethanol precipitation. Libraries were PCR-amplified for sequencing [22]. All reads generated from the CUT&Tag-seq of OLIG1 and OLIG2 were aligned to the mm39 mouse genome using Bowtie2 version 2.3.4. Sequence tags were aligned to the genome and subsequently analyzed by MACS2 software version 2.1.4 to detect genomic regions enriched for multiple overlapping DNA fragments (peaks), which were considered putative binding sites. Peaks with a false discovery rate lower than 5% were retained for further chromosomal region analysis. Visualization of peak distribution along genomic regions of genes of interest was performed using the Integrative Genomics Viewer (IGV).

### 2.10. Image Acquisition and Quantitative Analysis

Stained tissue sections were imaged using an Olympus VS120 microscope (Olympus Corporation, Tokyo, Japan) or an Olympus FV3000 (Olympus Corporation, Tokyo, Japan) confocal microscope system. Images were processed with Adobe Photoshop CC 25.0 and ImageJ 2.14.0 software without altering the original data.

For cell counting, 20-μm-thick sections from 3–4 mice were randomly selected at corresponding anatomical positions, and specific regions (1000 × 1000-pixel areas) were analyzed. Data analysis was performed using GraphPad Prism 8.0, Microsoft Excel 2021, and R v4.0.0. For individual experiments, samples from at least three control or mutant mice were examined. In morphological studies, multiple brain sections from comparable anatomical regions were analyzed. All analyzed mice were littermates with age- and sex-matched backgrounds.

Values and error bars represent mean ± SEM. The number of replicates (*n*) is indicated in the figures. *p*-values were determined using appropriate statistical tests. Statistical significance: * *p* < 0.05, ** *p* < 0.01, *** *p* < 0.001.

## 3. Results

### 3.1. OLIG1 and OLIG2 Exhibit High Co-Expression in Glioblastoma

**Figure 1:**
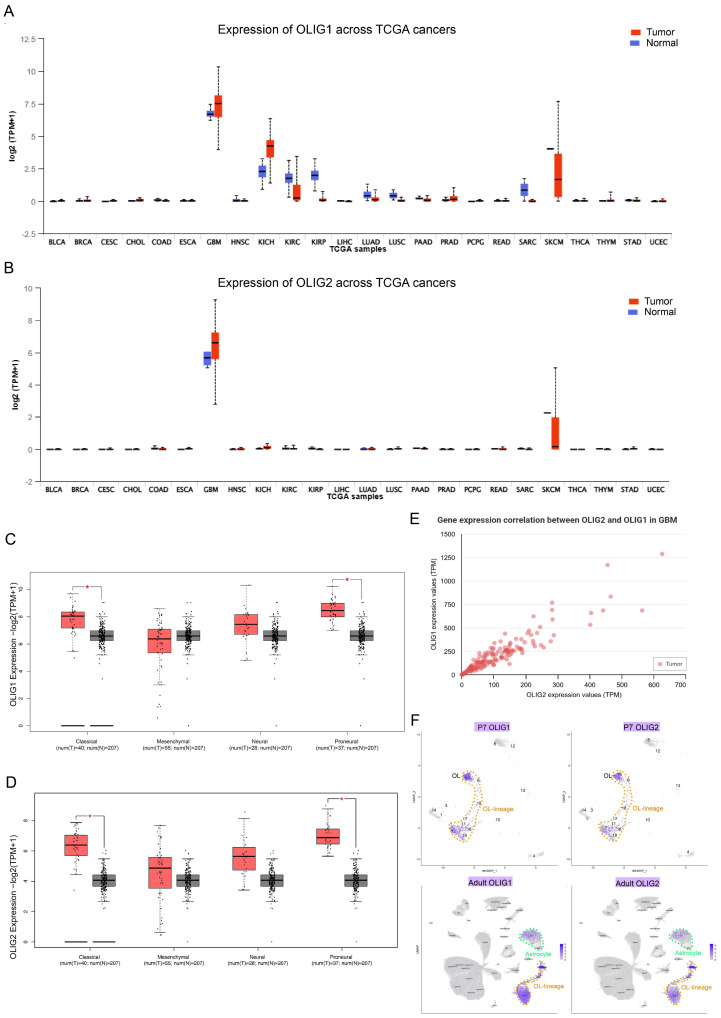
OLIG1 and OLIG2 exhibit high co-expression in human glioblastoma. (**A**) Boxplots represent log2 (TPM + 1) values for OLIG1 in distinct types of normal and tumor tissues according to the TCGA database. (**B**) Boxplots represent log2 (TPM + 1) values for OLIG2 in distinct types of normal and tumor tissues according to the TCGA database. (**C**) High OLIG2 mRNA expression in classical GBM (*n* = 40) in contrast to corresponding normal tissues (*n* = 207) from the GEPIA database, * *p* < 0.05. (**D**) High OLIG1 mRNA expression in classical GBM (*n* = 40) in contrast to corresponding normal tissues (*n* = 207) from the GEPIA database, * *p* < 0.05. (**E**) Correlation analysis of OLIG2 and OLIG1 expression in GBM from UALCAN. (**F**) UMAP of mouse P7 and adult cortical scRNA-seq data show OLIG2 and OLIG1 are co-expressed in oligodendrocyte lineage and astrocytes. BLCA, bladder urothelial carcinoma; BRCA, breast invasive carcinoma; CESC, cervical squamous cell carcinoma and endocervical adenocarcinoma; CHOL, cholangiocarcinoma; COAD, colon adenocarcinoma; ESCA, esophageal carcinoma; GBM, glioblastoma multiforme; HNSC, head and neck squamous cell carcinoma; KICH, kidney chromophobe; KIRC, kidney renal clear cell carcinoma; KIRP, kidney renal papillary cell carcinoma; LIHC, liver hepatocellular carcinoma; LUAD, lung adenocarcinoma; LUSC, lung squamous cell carcinoma; PAAD, pancreatic adenocarcinoma; PRAD, prostate adenocarcinoma; PCPG, pheochromocytoma and paraganglioma; READ, rectum adenocarcinoma; SARC, sarcoma; SKCM, skin cutaneous melanoma; THCA, thyroid carcinoma; THYM, thymoma; STAD, stomach adenocarcinoma; UCEC, uterine corpus endometrial carcinoma; TCGA, The Cancer Genome Atlas; TPM, transcripts per million reads.

To investigate the roles of OLIG1/2 in astrocytic GBM, we first examined their expression patterns in the human classical GBM subtype (astrocytic signature). First, we systematically evaluated the expression profiles of *OLIG1* and *OLIG2* across major tumor types using the TCGA database. The results revealed that both *OLIG1* and *OLIG2* are significantly overexpressed in GBM (Figure 1A,B). Analysis using GEPIA revealed that both *OLIG1* and *OLIG2* are expressed in all four subtypes of GBM, with significantly higher expression levels in the classical GBM subtype compared to normal tissues (*p* < 0.05) (Figure 1C,D). UALCAN analysis revealed that their expression levels exhibited a strong positive correlation (Pearson *r* = 0.92, Figure 1E), implying their potential co-regulation in shared biological processes within classical GBM. By integrating 10× genomics public scRNA-seq datasets from P7 and adult cerebral cortex, we observed the persistent co-expression of *Olig1* and *Olig2* in astrocytic lineage, suggesting their potential cooperative roles in astrocyte development (Figure 1F).

**Figure 2:**
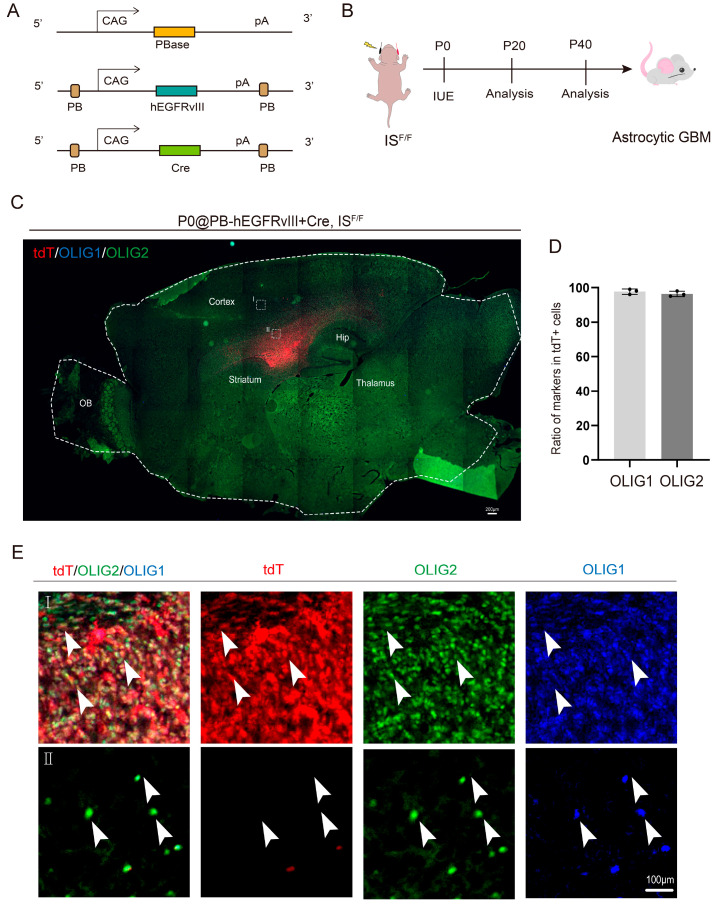
OLIG1 and OLIG2 are co-expressed in astrocytic GBM. (**A**) Schematic of the configuration of constructing plasmids for the mouse glioblastoma model. (**B**) Schematic of the workflow of constructing a mouse glioblastoma model. (**C**) Representative image showing the distribution of the tdT-positive and OLIG1/OLIG2-positive cells in the *IS^F/+^* GBM model mice at P40. (**D**) The statistical data show the percentage of OLIG1- and OLIG2-positive cells in the tdT-positive cells in SVZ of GBM model mice. *n* = 3 mice per group, mean ± SEM. (**E**) (I) Higher magnification of the boxed area in panel (CI) showing OLIG1 and OLIG2 co-express in the tdT-positive cells in the SVZ of *IS^F/+^* GBM model mice. (II) Higher magnification of the boxed area in panel (CII) showing OLIG1 and OLIG2 co-express in the tdT-negative cells in the *IS^F/+^* GBM model mice. The arrowheads indicate cells co-labeled with OLIG1/OLIG2/tdT (I) or OLIG1/OLIG2(II).

Next, we developed the astrocytic GBM mouse model in vivo. We first delivered plasmids encoding PiggyBac transposase (PBase), human *EGFRvIII* (*hEGFRvIII*), and *Cre* recombinase into the developing *IS^F/+^* reporter mouse cortex at P0 using in utero electroporation (IUE), subsequently examined at P20 and P40 (Figure 2A,B). *EGFRvIII*, a constitutively active mutation of epidermal growth factor receptor (EGFR) generated by an in-frame deletion of exons 2–7, is highly expressed in human glioblastoma and serves as a molecular hallmark of GBM [35]. Previous studies have demonstrated that electroporation of *EGFRvIII* in P0 mice recapitulates the pathological features of astrocytic GBM observed in humans [29]. The PiggyBac system ensures stable genomic integration, while *Cre* recombinase activates the tdTomato reporter (tdT+) in *IS^F/+^* mice, enabling the visualization of tumor cells.

At P40, the mice were analyzed, revealing that *PB-hEGFRvIII* successfully induced the formation of typical GBM lesions in the cerebral cortex (Figure 2C). This underscores the model’s ability to recapitulate key pathological features of GBM in vivo. Immunohistochemical analysis of tumor tissues in the subventricular zone (SVZ) demonstrated widespread co-expression of OLIG1 and OLIG2 in tdT+ tumor cells (Figure 2E). It is noteworthy that OLIG1 and OLIG2 are also co-expressed in the adjacent normal brain tissue (Figure 2E). Quantitative analysis confirmed that over 95% of tdT+ cells co-expressed both OLIG1 and OLIG2 (Figure 2D), validating their synergistic expression in astrocytic GBM model mice.

### 3.2. The Knockout of Olig1 and Olig2 in Astrocytic GBM Does Not Affect Astrocytic Characteristics

**Figure 3:**
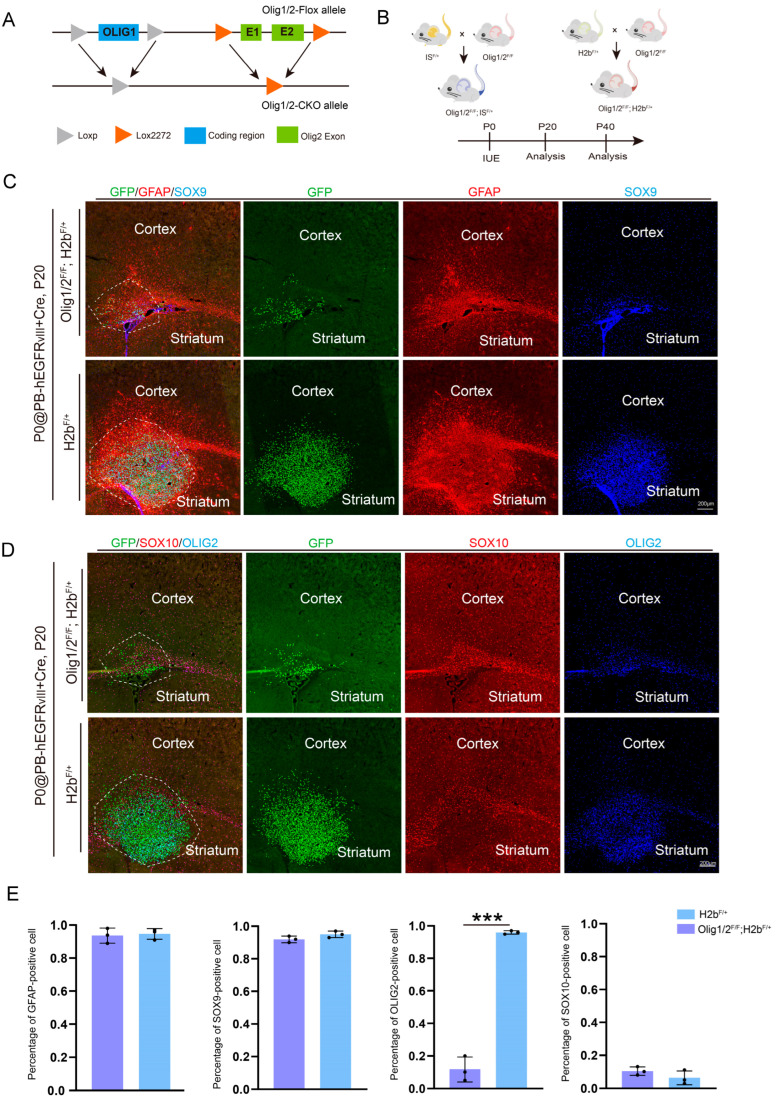
Knockout of *Olig1/2* does not alter glioma characteristics but inhibits glioma growth. (**A**) Schematic diagram of generating *Olig1/2* double knockout mice. (**B**) Schematic diagram of generating *Olig1/2^F/+^*; *IS^F/+^* and *Olig1/2^F/+^*; *H2b^F/+^* glioma model mice. (**C**) The immunostaining of the GFP, GFAP, and SOX9 in the coronal section of the *Olig1/2^F/+^*; *H2b^F/+^* and *H2b^F/+^* GBM model mice at P20. (**D**) The immunostaining of the GFP, SOX10, and OLIG2 in the coronal section of the *Olig1/2^F/+^*; *H2b^F/+^* and *H2b^F/+^* GBM model mice at P20. (**E**) Quantifications of the percentage of GFAP, SOX9, OLIG2, and SOX10-positive cells in GFP+ tumor cells in the position of the white dashed box in (**C**,**D**). *** *p* < 0.001. *n* = 3 mice per group.

To investigate the roles of *Olig1/2* in astrocytic GBM, we used a double conditional knockout mouse strain (*Olig1/2^F/+^*) [22]. Specifically, Lox2272 sites were inserted into non-conserved regions upstream of the 5′UTR and downstream of the 3′UTR of the *Olig2* gene, enabling the conditional knockout of exons 1 and 2 (Figure 3A). Through a secondary targeting strategy, Loxp sites were inserted 2 kb upstream of the 5′UTR and downstream of the 3′UTR of the *Olig1* gene, specifically targeting its single exon (Figure 3A).

Next, to achieve tumor cell-specific knockout of *Olig1/2* and enable labeling of the knockout cells, we first crossed *Olig1/2^F/F^* mice with two distinct reporter systems: *IS^F/+^* mice [28] (membrane-localized tdTomato labels the cell membrane) and *H2b^F/+^* mice [36] (nuclear-localized GFP marks the nucleus). This resulted in the generation of two mouse models: *Olig1/2^F/F^*; *IS^F/+^* and *Olig1/2^F/F^*; *H2b^F/+^* mice (Figure 3B).

At P0, plasmids encoding PBase, *hEGFRvIII*, and *Cre* were delivered into the control, *Olig1/2^F/F^*; *H2b^F/+^,* and *Olig1/2^F/F^*; *IS^F/+^* mice cortex via IUE (Figure 3B). The dual-reporter system, in combination with *Cre* recombinase, enables *Olig1/2* knockout in electroporated tumor cells while allowing visualization of the knockout cells and their progeny.

We first analyzed the *Olig1/2^F/F^*; *H2b^F/+^* mice at P20. Immunohistochemical staining revealed that GFP+ tumor cells in both control and *Olig1/2^F/+^*; *H2b^F/+^* groups exhibited robust expression of astrocytic markers, including GFAP and SOX9 (Figure 3C). Statistical data showed that almost all GFP+ cells express GFAP or SOX9 (Figure 3E). In the control group, GFP+ tumor cells exhibited high expression of OLIG2 but lacked the expression of the oligodendrocyte marker SOX10. Conversely, GFP+ cells in *Olig1/2^F/F^*; *H2b^F/+^* mice showed no co-labeling with OLIG2 or SOX10 (Figure 3D,E), confirming that the deletion of *Olig1/2* in tumor cells did not alter the astrocytic GBM cell phenotype.

### 3.3. Olig1/2 Knockout Reduced Tumor Growth Without Affecting Invasion

**Figure 4:**
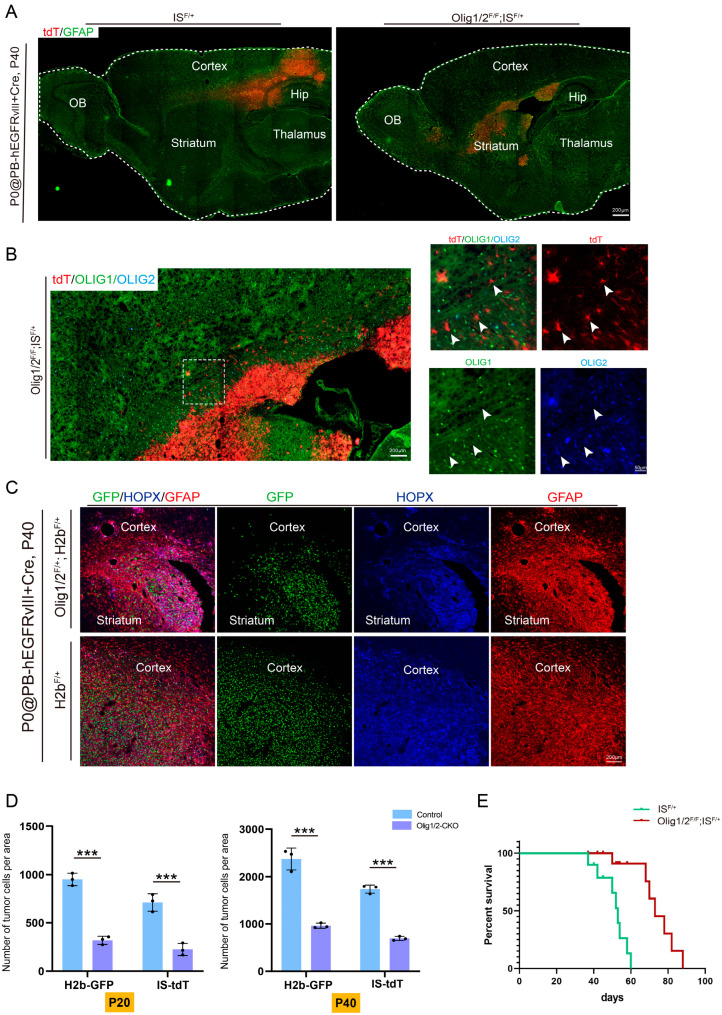
*Olig1/2* double knockout did not completely prevent tumor formation, but significantly reduced tumor size. (**A**) The immunostaining of the GFAP and tdT in the sagittal section of the *IS^F/+^* and *Olig1/2^F/+^*; *IS^F/+^* GBM mice at P40. (**B**) The immunostaining of the tdT, OLIG1, and OLIG2 in the *Olig1/2^F/+^*; *IS^F/+^* GBM mice at P40. White arrows indicate the non-co-localized cells. (**C**) The immunostaining of the GFP, GFAP, and HOPX in the coronal section of the *Olig1/2^F/+^*; *IS^F/+^* GBM mice at P40. (**D**) Quantifications of the tumor cells in the SVZ of *H2b* and *IS* mice at P20 and P40. *** *p* < 0.001. *n* = 3 mice per group. (**E**) Kaplan–Meier survival analysis of *IS^F/+^* (*n* = 10) and *Olig1/2^F/F^*; *IS^F/+^* (*n* = 14) GBM model mice.

Notably, a significant reduction in GFP+ tumor cells was observed in *Olig1/2^F/+^*; *H2b^F/+^* mice (Figure 3A). Given the significant reduction in GFP+ tumor cells in *Olig1/2^F/+^*; *H2b^F/+^* mice at P20, we further investigated whether *Olig1/2* knockout suppresses tumor initiation by systematically analyzing *Olig1/2^F/+^*; *H2b^F/+^* and *Olig1/2^F/+^*; *IS^F/+^* mice at a later stage (P40). Histological analysis revealed that tumors in *Olig1/2^F/+^*; *IS^F/+^* mice at P40 retained the capacity to initiate and migrate, infiltrating adjacent brain regions, albeit with reduced tumor volume (Figure 4A), indicating that *Olig1/2* knockout does not impair tumor initiation or migratory potential. Immunohistochemical staining confirmed a complete loss of OLIG1 and OLIG2 protein expression in tdT+ tumor cells of *Olig1/2^F/+^*; *IS^F/+^* mice (Figure 4B), validating the efficacy of the conditional knockout system in this study. The analysis of *Olig1/2^F/+^*; *IS^F/+^* tumor-bearing mice at P40 demonstrated significantly reduced tumor sizes compared to controls, while tumor cells maintained high expression of astrocytic markers GFAP and HOPX (Figure 4C).

To quantify the impact of *Olig1/2* knockout on tumorigenesis, we examined GFP+ (H2b model) or tdT+ (IS model) tumor cells in the control, *Olig1/2^F/F^*; *IS^F/+^*, and *Olig1/2^F/F^*; *H2b^F/+^* mice at both P20 and P40. The results showed that the number of tumor cells was significantly reduced in *Olig1/2* knockout groups compared to controls at both time points. Notably, a significant increase in tumor cells was shown in *Olig1/2* knockout groups from P20 to P40 (Figure 4D). Excitingly, compared to the control, the *Olig1/2*-ablated GBM mice had significantly extended survival times (Figure 4E). Collectively, these findings demonstrate that *Olig1/2* knockout does not affect tumor initiation but significantly impedes overall tumor growth.

### 3.4. Olig1/2 Reduced Tumor Growth by Inhibiting Glioma Cell Proliferation

**Figure 5:**
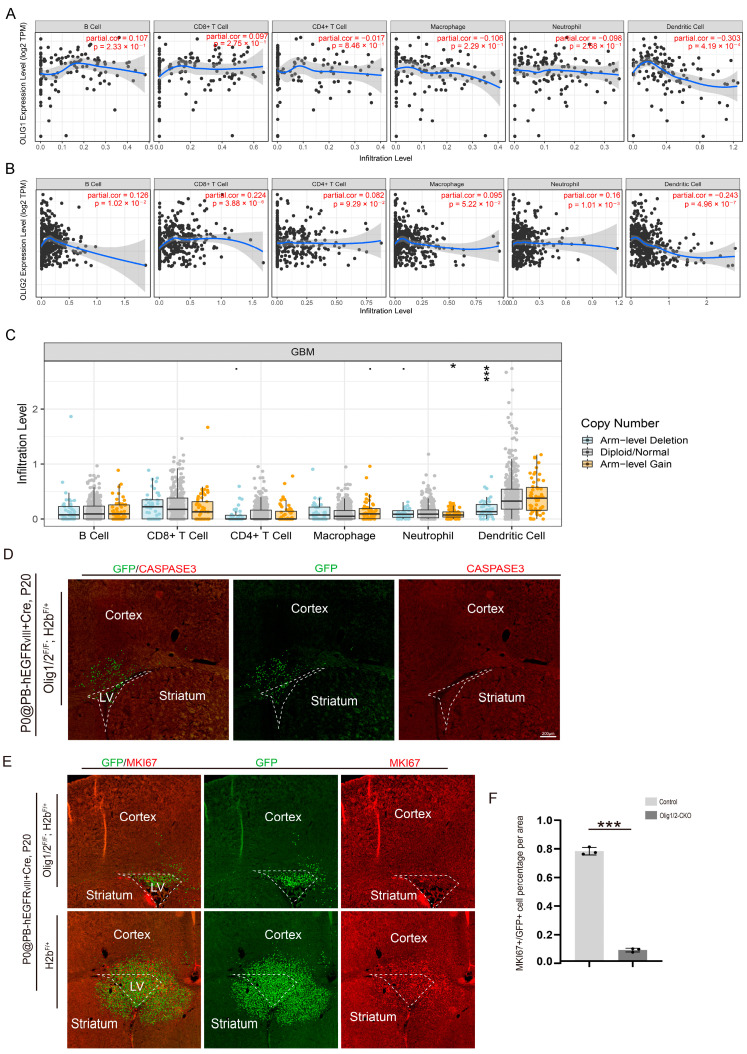
*Olig1/2* reduced tumor growth by inhibiting glioma cell proliferation. (**A**) The scatterplots show the correlation of OLIG2 expression with immune-infiltration level in GBM by TIMER. (**B**) The scatterplots show the correlation of OLIG1 expression with immune-infiltration level in GBM by TIMER. (**C**) The box plots show the comparison of tumor infiltration levels among GBM with different somatic copy number alterations for OLIG1 and OLIG2. The infiltration level is compared with the normal using a two-sided Wilcoxon rank sum test, * *p* < 0.05, *** *p* < 0.001. (**D**) The immunostaining of the GFP and CASPASE3 in the coronal section of the *Olig1/2^F/+^*; *H2b^F/+^* GBM mice at P20. (**E**) The immunostaining of the GFP and MKI67 in the coronal section of the *Olig1/2^F/+^*; *H2b^F/+^* and *H2b^F/+^* GBM mice at P20. (**F**) The statistical data show the percentage of MKI67-positive cells in the GFP-positive cells in the SVZ of control and *Olig1/2-CKO* GBM mice. *n* = 3 mice per group, *** *p* < 0.001, mean ± s.e.m.

To investigate if the reduction in tumor cells without *Olig1/2* resulted from the altered microenvironment, we investigated the correlation between the expression levels of OLIG1 and OLIG2 and the abundance of six tumor-infiltrating immune cell types in GBM by TIMER. The scatter plot detailed that the expression of OLIG2 level is not associated with immune cells such as B cells (r = 0.126), CD8+ T cells (r = 0.224), CD4+ T cells (r = 0.082), macrophage (r = 0.095), neutrophil (r = 0.16), or CD4+ T cells (r = −0.243). The expression of OLIG1 level is also not associated with B cells (r = 0.107), CD8+ T cells (r = 0.097), CD4+ T cells (r = −0.017), macrophage (r = −0.106), neutrophil (r = −0.098), and CD4+ T cells (r = −0.303) (Figure 5A).

Next, we explored the correlation between somatic copy number alterations and immune-infiltration abundance in GBM. Since *Olig1* and *Olig2* are co-located on chromosome 21q, we utilized the TIMER method to analyze immune infiltration following arm-level deletions. The statistical plot indicated no correlation between OLIG1/2 expression and 5 immune cell types, with only dendritic cells showing a negative correlation. Notably, OLIG1/2 are key transcription factors for oligodendrocyte differentiation. A copy number gain in these genes may accelerate myelin regeneration, restore neuronal electrical signaling, and reduce persistent inflammatory damage, thereby indirectly lowering the demand for neutrophil infiltration (Figure 5C). These findings suggested that the slowed tumor cell growth following *Olig1/2* deletion is not attributable to alterations in the tumor microenvironment.

We next investigated whether this phenomenon is driven by intrinsic changes in tumor cell apoptosis or proliferation. We performed an apoptosis analysis by staining *Olig1/2^F/+^*; *H2b^F/+^* brain tissues at P20 with CASPASE-3, a marker commonly used for the detection of cell apoptosis [37]. The results showed that no detectable CASPASE-3+ signals in GFP+ tumor cells were observed after *Olig1/2* knockout (Figure 5D), indicating that the deletion of *Olig1/2* did not trigger tumor cell death. Subsequent staining of MKI67, a well-established marker of proliferation [38], revealed a marked reduction in MKI67 co-labeling within GFP+ tumor cells of the knockout group compared to the controls (Figure 5E,F). This indicates that the absence of *Olig1/2* leads to a downregulation of MKI67 expression, which means the reduced tumor growth is caused by slowed tumor proliferation.

### 3.5. OLIG1/2 Promote Cell Proliferation by Binding to Various Cyclins

**Figure 6:**
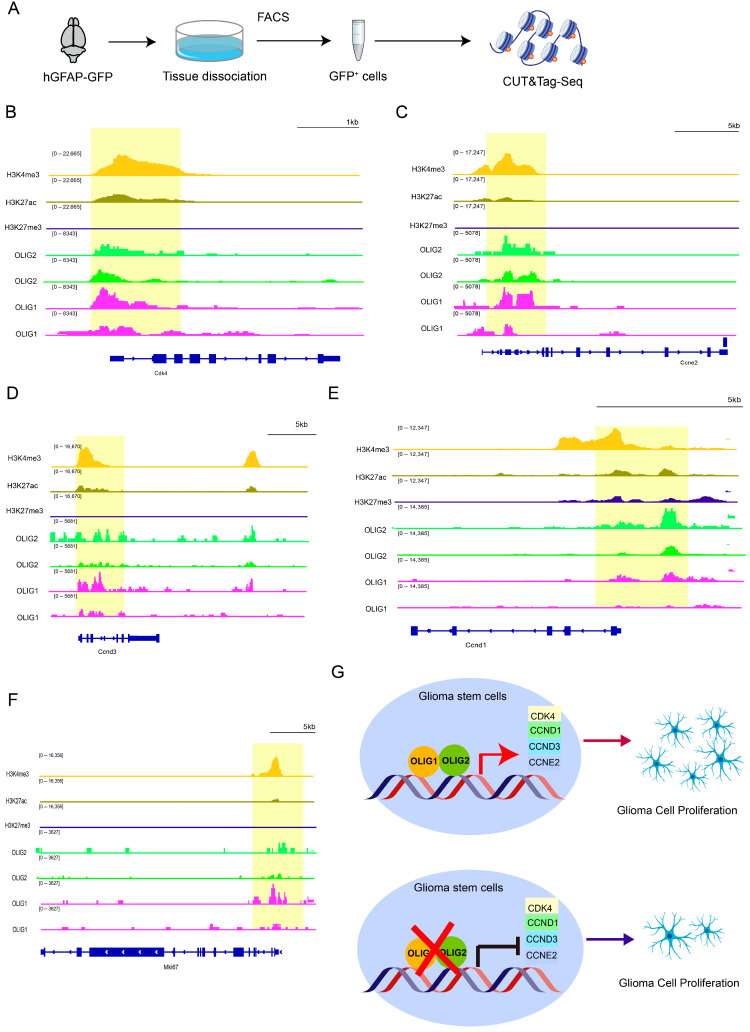
OLIG1/2 co-bind to the various cyclins to regulate proliferation. (**A**) The experimental workflow of the CUT&Tag assay. (**B**) Integrative Genomics Viewer (IGV) snapshot showing that OLIG1 and OLIG2 bind to the promoter of *Cdk4* (highlighted by the yellow box). Replicates of OLIG1 and OLIG2 CUT&Tag data from GSE273171 [22], CUT&Tag tracks using H3K27ac, H3K4me3, and H3K27me3 antibodies for *Mki67* are shown. (**C**) IGV snapshot showing that OLIG1 and OLIG2 bind to the promoter of *Ccne2* (highlighted by the yellow box). (**D**) IGV snapshot showing that OLIG1 and OLIG2 bind to the promoter of *Ccnd3* (highlighted by the yellow box). (**E**) IGV snapshot showing that OLIG1 and OLIG2 bind to the promoter of *Ccnd1* (highlighted by the yellow box). (**F**) IGV snapshot showing that OLIG1 and OLIG2 bind to the promoter of *Mki67* (highlighted by the yellow box). (**G**) The schematic model demonstrates that *Olig1/2* cooperatively drive tumor cell proliferation through transcriptional co-regulation of cell cycle regulators.

Given that many developmental programs are reused during malignancy, to further validate the mechanism by which *Olig1/2* regulate cell proliferation, we analyzed the published CUT&Tag data for OLIG1 and OLIG2, along with histone modification profiles, from E18.5-P1 *hGFAP-GFP* mouse cerebral cortex (GSE273171) [22] (Figure 6A). As expected, we observed prominent binding peaks of OLIG1 and OLIG2 at the promoter region of *Mki67* (Figure 6F), which was concurrently enriched with the activating histone marker H3K4me3 (Figure 6E). Furthermore, we discovered that OLIG1 and OLIG2 co-bind to the promoters of a series of core cell cycle promoters, including *Cdk4*, *Ccne2*, *Ccnd3*, and *Ccnd1* (Figure 6B–E), genes critical for G1/S phase transition and cyclin-dependent kinase activation [39,40]. These binding sites are also co-occupied by histone H3K4me3 modifications, suggesting OLIG1 and OLIG2 co-promote the cell cycle by targeting these genes, thereby influencing cell proliferation.

These findings position *Olig1/2* as master regulators bridging developmental gliogenesis and oncogenic proliferation. By co-opting chromatin-modifying complexes to activate cyclin-driven cell cycle programs, *Olig1/2* may license neural precursor-like proliferative states in glioma stem cells—a potential vulnerability for targeted therapies (Figure 6G).

## 4. Discussion

The present study unveils a critical role for *Olig1/2* as synergistic drivers of astrocytic GBM proliferation through direct transcriptional activation of various cyclins, while maintaining tumor cell identity. Our findings bridge a significant gap in understanding how these transcription factors, traditionally associated with oligodendroglial biology, contribute to astrocytic-featured GBM pathogenesis.

The transcription factors *Olig1* and *Olig2* exhibit a high degree of structural homology and coordinated expression patterns in the central nervous system, suggesting potential functional overlaps. Early studies of *Olig1* and *Olig2* primarily focused on their roles in oligodendrocyte lineage development. Previous research has demonstrated that *Olig2* is essential for the specification of spinal cord oligodendrocyte progenitor cells (OPCs) [41,42]. In contrast, *Olig1* deficiency only delays OPC maturation, with residual PDGFRA-positive cell populations persisting in the forebrain [43]. Notably, dual knockout of *Olig1/2* completely eliminates OPCs [8,44], indicating functional redundancy during specific developmental stages. Beyond their roles in the oligodendrocyte lineage, emerging evidence highlights the critical roles of *Olig1* and *Olig2* in astrocyte development [45,46,47,48]. Recent studies have revealed that OLIG2 and ASCL1 bind to each other’s genomic loci and downstream targets, cooperatively regulating tumor cell plasticity and heterogeneity [49], suggesting that transcription factor gene combinations—rather than individual factors—may drive tumor progression.

Both *Olig1* and *Olig2* are ubiquitously expressed in gliomas and play pivotal roles in tumorigenesis and phenotypic plasticity [14,20,50]. Recent work showed that the loss of PTEN/p53 in *Olig1/2*-expressing intermediate progenitors induces gliomagenesis with distinct therapeutic vulnerabilities [24]. Furthermore, *Olig2* exerts broad and critical functions across glioma subtypes [13,20]. In a predisposed glioblastoma animal model, ablation of mitotic OLIG2-positive progenitor cells halted tumor growth, identifying these progenitors as seeds for glioma propagation [17]. Additional studies demonstrated that *Olig2* deletion reduces tumor growth and drives a phenotypic shift from oligodendroglial to astrocytic features, concomitant with PDGFRA downregulation and compensatory EGFR signaling pathway activation, revealing alternative routes for tumor recurrence [20]. In patient-derived glioma stem cells (GSCs), *Olig2* knockdown suppresses PDGFRA, while *Olig2* silencing induces transcriptional reprogramming across GSC lines—transitioning from proneural to classical GBM expression patterns or proneural-to-mesenchymal transformation—with context-dependent regulation of EGFR expression by *Olig2* [20].

While *Olig2* has been extensively studied in proneural GBM subtypes for its role in maintaining glioma stem cells (GSCs), our work highlights the cooperative function of *Olig1/2* in astrocytic-featured GBM. The robust co-expression of *Olig1/2* in both human GBM and our mouse model (Figure 1B–E and Figure 2C,D) suggests their partnership extends beyond oligodendroglial contexts. Notably, their persistent co-expression during astrocytic lineage development (Figure 1F) implies an evolutionarily conserved mechanism co-opted in tumorigenesis. This challenges the conventional view that *Olig1/2* are exclusively oligodendrocyte-lineage regulators and positions them as versatile oncogenes adaptable to diverse gliomal niches.

Our mechanistic dissection identifies various cyclins as a direct transcriptional target of *Olig1/2* (Figure 6B–E). The enrichment of *Olig1/2* binding and H3K4me3 at the *Cdk4*, *Ccne2*, *Ccnd3*, and *Ccnd1* promoters suggests cooperative regulation of transcriptional activation. The identified target genes (*Cdk4*, *Ccne2*, *Ccnd3*, and *Ccnd1*) are core regulatory factors of the cell cycle that drive the transition from the quiescent phase (G1) to the DNA synthesis phase (S), and they are not only essential for neuroglial development but are also frequently dysregulated in gliomas. This dual role aligns with the concept of “onco-exaptation” where developmental transcription factors hijack proliferative pathways in malignancy.

Notably, although *Olig1/2* knockout significantly suppresses tumor proliferation, persistent tumor formation is still detectable in our model (Figure 5). This phenomenon may be closely associated with the introduction of constitutively active EGFR mutations (hEGFRvIII) in the model. EGFRvIII likely bypasses the proliferative inhibition caused by *Olig1/2* loss through constitutive activation of downstream signaling pathways (e.g., PI3K/AKT/mTOR and MAPK/ERK), thereby providing alternative survival and proliferative signals for tumor cells. This compensatory activation mechanism aligns with prior studies. In *Olig2*-deficient glioma models, the downregulation of PDGFRA signaling is accompanied by feedback upregulation of the EGFR pathway to sustain tumor growth [20].

This finding carries critical implications for clinical therapeutic strategies. Interventions targeting a single molecular node (e.g., *Olig1/2*) may exhibit limited efficacy due to signaling pathway redundancy in tumor cells. Thus, combining *Olig1/2* inhibition with EGFR pathway blockade (e.g., using EGFR-TKIs such as erlotinib) [51] or targeting shared downstream effectors may more effectively suppress compensatory proliferation. Importantly, compensatory mechanisms may vary across GBM subtypes—mesenchymal GBMs with NF-κB pathway activation may exhibit enhanced EGFR-independent resistance, while proneural subtypes may rely more on PDGFRA-mediated compensatory signaling [13]. Future studies should systematically dissect dynamic signaling rewiring following *Olig1/2* loss in patient-derived xenograft models to inform precision combination therapies.

## Data Availability

The data presented in this article are available.
